# Supplementary material for: Reliability of online, remote neuropsychological assessment in people with and without subjective cognitive decline
Source: PLOS Digit Health. 2025 Apr 8;4(4):e0000682. doi: 10.1371/journal.pdig.0000682 (PMC11977984; doi:10.1371/journal.pdig.0000682)
Supplement: Attachment — Submitted filename: Response_to_Reviewers.docx [file pdig.0000682.s002.docx]

The authors wish to thank all reviewers for their time and their helpful feedback which we feel has considerably improved the manuscript.

Reviewer #1: Thank you for letting me read this important piece of work.

The manuscript is well-written and comprehensively explains the methodology. The study design is appropriate for this research question and hypothesis. The results are well explained however since statistical analysis is not my area of expertise, I hope the editors have sent this for review to those who do.

The limtations have been explicitly stated in the discussion section and the conclusions are modest. Therefore, there are no suggestions for revisions from my side.

I wish the authors best of luck.

We thank Reviewer #1 for their feedback.

Reviewer #2: The aim of the research is interesting, but the methodology is not completely justifiable. For example, the statistical analysis relating to the age differences of the two experimental groups involved could have been avoided by adopting homogeneous groups from this point of view, in this way the results would have been cleaner.

We thank Reviewer #2 for their feedback. We used an opportunistic, community-based recruitment approach as this methodology is in line with the clinical question. This meant groups were not matched for age during the recruitment process. We have added a sentence to state this explicitly within the manuscript (page 13).

The conclusions are not fully supported by the data, which show inconsistencies that, although not significant, are in contrast with results.

Thank you for highlighting this. We have tempered the conclusions in response to your and Reviewer #3’s feedback (page 28).

Furthermore, the remote test does not allow to completely control the experimental conditions nor the neuropsychological characteristics of the participants, who must simply be "trusted" without a clinical opinion.

Thank you for this important point. We have further emphasised this limitation on page 27, with the clarification “meaning SCD could not be clarified by a clinician”.

Reviewer #3: The paper investigates the reliability of an online-administered suite of cognitive tests on individuals with and without SCD and whether these test measures and changes in the measures show significant group differences. The paper is written well and statistically sound. However, there are some major concerns which the paper needs to address.

We thank Reviewer #3 for their feedback.

1. The main claim from the paper, which is stated in multiple places, e.g., "The results suggest online, remote neuropsychological assessment is a promising option for assessing and monitoring SCD." from the abstract, is not supported by the study and findings. The results show that of the seven tests, only the SART and picture recognition showed moderate test-retest reliability across groups (the picture recognition task barely making into the moderate category). None of the measures, or their changes, showed significant group differences. Uncorrected, rather the measures showed the opposite effect, with SCD reporting higher cognitive scores. As such, the paper cannot claim the results show any promising results from the suite to assess or monitor SCD. The abstract, intro, and conclusions should be edited accordingly. As it stands currently, the main takeaway from the paper is that from a suite of deployed tests, one or two tests show moderate test re-test validity in SCD/non-SCD population.

Thank you for your important feedback. We have edited these sections to highlight the need to ensure reliability and sensitivity of tests: see changes to page 2, page 3, conclusion page 28.

2. The re-test is scheduled six months after the baseline. The choice for this gap in the study design is not explained. Why were the CCI scores not obtained in the follow-up? Probably the group composition of SCD/Non-SCD changed at follow-up. What is the impact of such possible change on the pursued analysis?

Thank you for these points. In the present study, we used CCI scores to group the participants at the baseline assessment. We then explored differences in baseline and longitudinal change in Neuron test performance between the groups. However, we agree that re-assessment using the CCI may reveal changes in the group composition at follow-up which may be useful to explore. We have added this as a limitation: “A further limitation was the decision to not re-assess CCI scores at follow up, which were instead used for classification at baseline assessment. Doing this would have allowed us to understand whether the group composition (SCD, Non-SCD) had altered at the follow up.” (page 27).

The following justification for the use of a six-month follow-up period has been added to the manuscript: “A six-month delay between baseline and follow-up was chosen to be consistent with typical assessment schedules used to monitor cognitive changes in clinical practice (e.g. [38]).” (page 10).

3. The paper refers to their earlier work in [A] and positions that the current work expands on the suite of considered tests for the analysis. In [A], several tests were deployed in the remote test: Reaction Time task, Trail Making Test -A, Trail Making Test -B, Picture Recognition, Spatial Span Backwards, Go/No-Go test, Fragmented Letters, and Virtual Supermarket Test. The current paper only mentions that limited (four) tests were deployed. Am I missing something? Why were some of the tests mentioned in [A] not included in this work?

Thank you for raising this. We agree that this was unclear in the previous version of the manuscript. The section referring to the tests used in [A] (page 7) refers to the four subtests which were evaluated for one-week test-retest reliability in the paper (not all of the tests mentioned in [A] were repeated for the test-retest reliability analysis within that paper). To make this clearer we have changed the text to clarify that four subtests were repeated (rather than four tests) in [A] (page 7). In the present study, we evaluated test-retest reliability for selected tests relevant to SCD (i.e. tests which measure cognitive abilities commonly affected in early stages of dementia) and for a clinically relevant time period of six-months follow-up. The choice of test selection was previously explained on page 12 (unchanged), however we have added “relevant to SCD” in the section on page 7 to further clarify the rationale for the selection of tests used.

Other Issues:

1. In the Introduction, please cite tests when they are first mentioned.

Thank you for this point. A citation for Trail Making Test B has been added to page 5.

2. In the Introduction, this statement is made: "It is unclear whether online neuropsychological tests, completed remotely and unsupervised, show comparable psychometric properties to the 'gold-standard' in-person pen and paper tests." However, this paper does not address this question.

Thank you for pointing this out. This statement has been edited to remove reference to comparability with in-person tests (page 5).

3. In Methods, I am not sure if mentioning developer's name is any helpful or informative and might be rather distracting for the scientific message that the paper intends to convey. Reference/Citation to existing tools should suffice.

Thank you for this suggestion – references to developers have been removed (various sentences on page 7).

4. In Methods, please avoid very generic and marketing-sounding messages like "Some standardised data are available and new tests are being developed."

Thank you for this suggestion. This sentence has been removed (page 7).

5. In Methods, the statement "Test-retest reliability has been evaluated for a selection of the NeurOn tests (Reaction
Time, a Go-No/Go test and the Virtual Supermarket Task)" does not list four tests as is mentioned in the next sentence.

Thank you for highlighting this. The sentences have been edited to clarify that four subtests were previously investigated (page 7).

6. In the Discussion, "There was poor to good reliability across all outcome measures." is also very generic and could probably be said for any randomly deployed suites of the test. Some specifics would be helpful.

Thank you for this suggestion. We have added the following to page 25:

“Picture Recognition showed the best evidence for test-retest reliability for the Non-SCD group, with moderate to good reliability for all three outcome measures. Whereas, Digit Span Backwards and the Sustained Attention to Response Test showed good evidence for test-reliability for the SCD group, with two and three of the outcome measures showing at least moderate reliability, respectively.”

[A] Morrissey, Sol, Rachel Gillings, and Michael Hornberger. "Feasibility and reliability of online vs in-person cognitive testing in healthy older people." Plos one 19, no. 8 (2024): e0309006.
